# Unraveling research self-efficacy and concerns as factors associated with psychological distress among nursing scholars in the era of artificial intelligence: a multi-campus survey

**DOI:** 10.1186/s12912-025-03353-w

**Published:** 2025-07-01

**Authors:** Mohamed Hussein Ramadan Atta, Mohamed Zoromba, Hussein M. Magdi, Heba El-Gazar, Shimmaa Mohamed Elsayed, Omnya Sobhy Mohamad El-ayari, Selwan Mahmoud Ibrahim Balha, Alzahraa Abdel Aziz Omar Abdel Rahman, Rasha Salah Eweida

**Affiliations:** 1 Psychiatric and Mental Health Nursing Department, Faculty of Nursing, Alexandria University, Alexandria City, Egypt; 2 Nursing College, Prince Sattam Bin Abdulaziz University, Kharj City, Saudi Arabia; 3 Mental Health Nursing, Faculty of Nursing, Mansoura University, Mansoura, Egypt; 4 Psychiatric and Mental Health Nursing, Faculty of Nursing, Beni-Suef University, Beni-Suef, Egypt; 5 Administration Department, Faculty of Nursing, Port Said University, Port Said City, Egypt; 6 Critical Care and Emergency Nursing, Faculty of Nursing, Damnhour University, Damnhour, Egypt; 7 Psychiatric Nursing and Mental Health, Faculty of Nursing, Kafr Elsheikh University, Kafr Elsheikh City, Egypt; 8 Psychiatric and Mental Health Nursing, Faculty of Nursing, Tanta University, Tanta, Egypt; 9 Psychiatric Mental Health Nursing, Faculty of Nursing, Minya University, Minya City, Egypt; 10 Psychiatric and Mental Health Specialty, Nursing Department, College of Health and Sport Sciences, University of Bahrain, Manama, Bahrain

**Keywords:** Artificial intelligence, Concerns, Psychological distress, Self-efficacy, Scholars

## Abstract

**Background:**

The relentless march of artificial intelligence (AI) has emerged as a formidable catalyst, offering a toolbox of novel tools and methodologies with the potential to revolutionize the very essence of research practices.

**Objectives:**

To explore the association of levels of research self-efficacy and concerns related to artificial intelligence with psychological distress among nursing scholars.

**Methods:**

A descriptive, multi-campus survey, cross-sectional design was adopted in this study. The study employed a clustered sampling technique to ensure representation from different regions. A sample of 1494 nursing scholars completed the nursing scholars’ concerns toward the artificial intelligence questionnaire, Kessler psychological distress, and the research self-efficacy scale.

**Results:**

Psychological distress is negatively correlated with researchers’ self-efficacy while positively correlated with concerns regarding AI. Concerns about AI contributed to increased psychological distress. In addition, female academic staff reported significantly higher psychological distress compared to males, and those younger staff members experienced more distress than older colleagues, and lower research self-efficacy was associated with higher psychological distress, and those with less experience in publishing and fewer published articles tended to report more distress.

**Conclusion:**

This study underscores the critical role of research self-efficacy in the era of artificial intelligence in mitigating psychological distress, highlighting its significance as a key protective factor. These insights contribute to a deeper understanding of the factors influencing psychological well-being in academic and research settings, guiding future strategies to foster resilience and mental well-being.

**Clinical trial registration:**

Not applicable.

**Supplementary Information:**

The online version contains supplementary material available at 10.1186/s12912-025-03353-w.

## Introduction

The relentless march of artificial intelligence (AI), marked by its ability to emulate human-like cognitive processes, problem-solving capabilities, and the capacity for autonomous learning and self-correction [1, 2], has ushered in a transformative era across various domains of science and technology [3]. In this epoch, scholars and researchers find themselves navigating the shifting landscape of AI’s influence, where innovation and uncertainty are inextricably intertwined [4, 5].

While AI has emerged as a formidable catalyst, offering a toolbox of novel tools and methodologies with the potential to revolutionize the very essence of research practices [6], it also brings forth a unique set of challenges and complexities that researchers must navigate [7]. Among these challenges lie scholars’ concerns related to AI, which have become significant barriers to the widespread adoption of AI technologies [8]. Scholars continue to voice their apprehensions, raising valid questions about ethical considerations, the transformation of traditional research methodologies, and the societal impact of AI [9]. These concerns not only pose considerable challenges but also have the potential to manifest as psychological health-related problems.

The growing integration of AI into healthcare has significantly impacted the nursing profession, influencing clinical decision-making, patient care, and professional identity (3,6). AI-driven technologies, such as predictive analytics, virtual health assistants, and automated documentation systems, are increasingly being utilized to enhance efficiency and patient outcomes (7). However, alongside these advancements, nurses face ethical dilemmas, concerns about job displacement, and challenges in adapting to AI-driven workflows (8,9). These uncertainties can contribute to psychological stress, affecting nurses’ well-being and professional confidence. Understanding scholars’ concerns about AI is particularly relevant to nursing, as it informs strategies to address barriers to AI adoption, integrate AI education into nursing curricula, and support nurses in navigating technological transformations within healthcare settings (4,5).

Remarkably, despite the burgeoning discourse on AI-related concerns [10], there remains a conspicuous gap in the research landscape. To our knowledge, no study has ventured to investigate the direct association between scholars’ concerns about AI and the psychological distress they may experience as a result. This gap is significant, as it leaves uncharted territory in understanding the intricate psychological ramifications of the AI era.

Furthermore, this study extends its scope to explore the connections between personality factors, specifically self-efficacy [3], and psychological distress. Self-efficacy, defined as “the belief in one’s capabilities, is a cornerstone upon which individual well-being is built” [11]. Therefore, this study aspires to unravel the association between researchers’ self-efficacy, concerns related to AI, and the resulting psychological distress they may experience. This, by virtue, would establish an axiom framework with which to frame a potentially nuanced relationship.

## Literature review

Self-efficacy refers to the judgments individuals hold about their abilities to successfully perform courses of action to achieve given goals [12]. Consequently, self-efficacy contributes to a sense of competence and capability for success [13]. Scholars’ self-efficacy holds significant importance in academic settings [14]. Therefore, scholars possessing a heightened sense of self-efficacy are more inclined to initiate self-control behaviors and exhibit reduced academic procrastination [15]. Additionally, heightened self-efficacy may foster increased awareness and a positive attitude regarding AI [16].

The utilization of AI in academic research has already permeated various aspects of the research process, including paper writing, language translation, the assessment of written work, text summarization, plagiarism detection, and the advent of automated peer-review platforms [17]. However, despite the evident positive utilization and benefits scholars derive from the application of AI, there is a concurrent rise in scholars’ concerns regarding its use. These concerns encompass ethical issues, the dearth of accumulated data, as well as the potential for distortion and bias in information [16]. Scholars’ concerns related to AI can shape their attitudes toward this technology [18] and may even lead to psychological challenges [19]. One such challenge is the onset of psychological distress.

Psychological distress is defined as the discomfort individuals may experience when they find it challenging to cope with various life problems or encounter difficulties in their daily lives. It represents a state characterized by emotional unease and unpleasant feelings [20]. This issue is a prevalent concern in healthcare worldwide, particularly among academic professionals [21]. Psychological distress can be influenced by a wide range of antecedents, encompassing environmental factors, work-related factors, personal life factors, and social factors [22]. While it is often considered a temporary state, it can have lasting and significant consequences if not effectively addressed [21]. Individuals experiencing psychological distress are at an increased risk of developing affective disorders, exhibiting problematic work behaviors, and delivering lower-quality work [23].

Despite extensive research on research self-efficacy and its impact on academic performance, research productivity, and psychological well-being [24, 25], there remains a critical gap in understanding how scholars’ concerns about AI influence their psychological distress, particularly in the context of nursing research. Previous studies have explored self-efficacy about online learning readiness [26], and its role in buffering stress, anxiety, and depression [27, 28]. However, no study has specifically examined the intersection of research self-efficacy, concerns about AI, and psychological distress among nursing scholars [10]. In the rapidly evolving landscape of AI-driven research, it is imperative to understand how concerns about AI contribute to psychological distress and whether self-efficacy serves as a protective factor. Our study is the first to address this gap, providing novel insights into the psychological impact of AI concerns on nursing scholars and emphasizing the need for targeted interventions to enhance self-efficacy, alleviate distress, and foster resilience in the AI era. Therefore, understanding the factors associated with psychological distress is crucial for developing interventions that can support the mental well-being of scholars.

### Objectives of this study

The primary objective of this study was to explore the association of levels of research self-efficacy and concerns related to artificial intelligence with psychological distress among nursing scholars.

### Research question

What is the association between the level of research self-efficacy, concerns related to artificial intelligence, and psychological distress among nursing scholars?

### Study methods

#### Design

The study utilized a descriptive, cross-sectional design, adhering to the Strengthening the Reporting of Observational Studies in Epidemiology (STROBE) guidelines, which occurred between July and September 2023.

#### Sample and setting

**Fig. 1:**
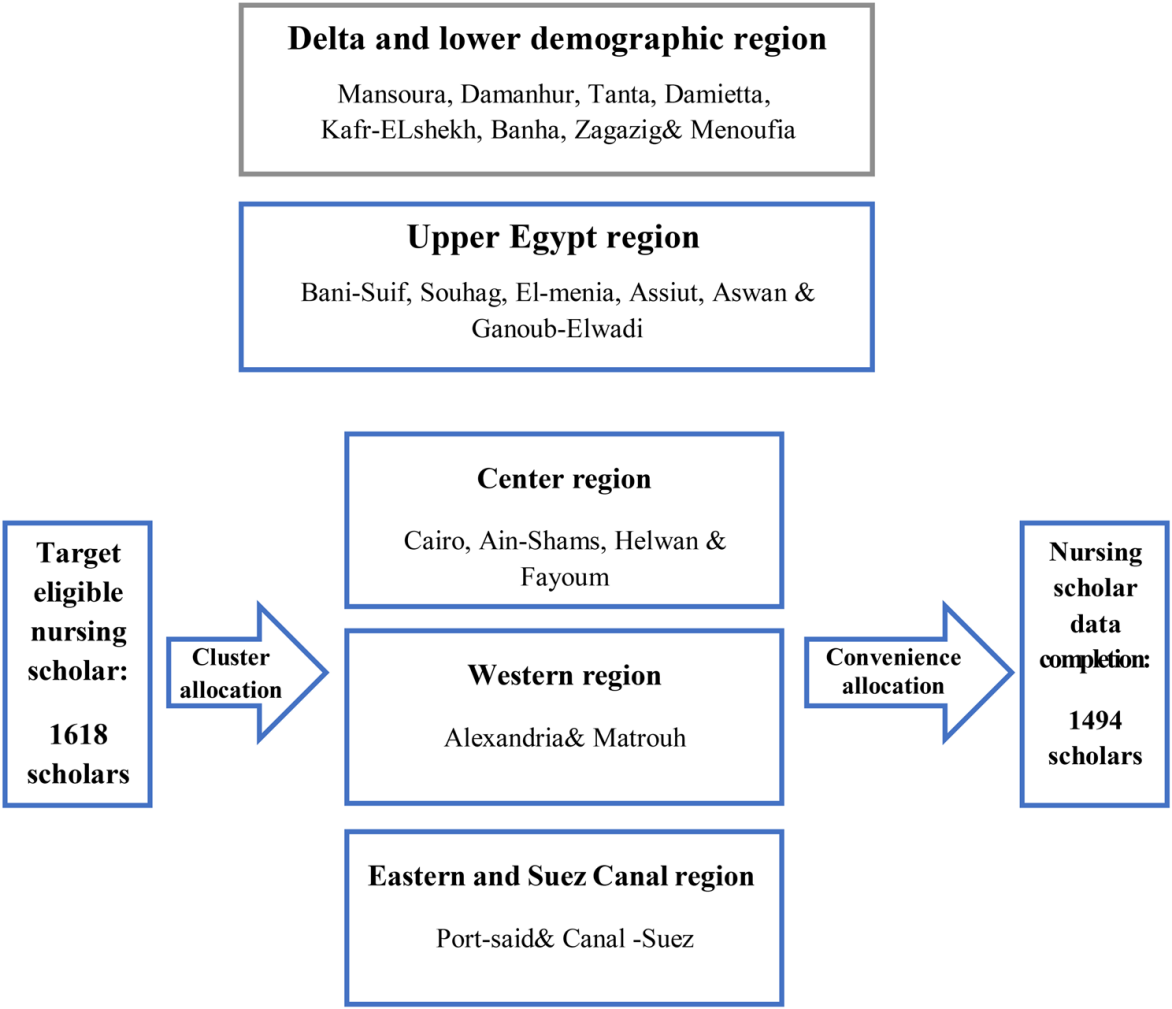
Study flow graph

The sample size for this study was determined using MedCalc 15.8 (https://www.medcalc.org/) and included 1,494 researchers. To ensure adequate representation, a clustered sampling technique was employed across 22 governmental nursing faculties in Egypt. These faculties were distributed across five geographical regions: Delta and Lower Egypt, Central Region, Western Region, Eastern and Suez Canal Region, and Upper Egypt (30) (Fig. 1). Egypt has a total of 25 governmental nursing faculties, meaning that the study captured 88% of these institutions, ensuring broad regional representation.

To determine the required sample size, an internal pilot study was conducted on 100 participants, revealing that 36.0% exhibited high to very high psychological distress. Using this estimate, the sample size calculation was based on a 5.0% alpha error, 80.0% study power, and 5.0% precision. These parameters were chosen to ensure statistical rigor and minimize both Type I and Type II errors while achieving an accurate estimation of psychological distress prevalence among researchers. The pilot study findings were not used to establish a causal relationship but rather to inform an appropriate sample size for meaningful statistical analysis in the current study.

### Instruments

The survey questionnaire consisted of four parts: Section A collected information on socio-demographic & academic data such as age, gender, and affiliation. Section B assessed Nursing Scholars’ Concerns Toward Artificial Intelligence (Appendix 1). This tool was developed by researchers based on a review of relevant literature. Fifteen experts revised the tool to assess content validity, completeness, item clarity, and the items’ relevance for the measured construct. The appropriate modifications were made accordingly. The tool consists of 20 items intended to assess researchers’ concerns about using AI in research. Each item was rated on a five-point Likert scale. The responses range from “all the time” (score 5) to “none of the time” (score 1), with higher scores indicating an advanced concern level toward using AI in research. The tool was tested for validity and reliability. The content validity index (CVI) of clarity and relevance for each item ranged from 0.68 to 1. The majority score of clarity and relevance equals 0.88 for most of the items. CVI of experts ranged from 0.84 to 0.94, and the total content validity index equaled 0.90. Adding to that, this tool exhibits an even higher Cronbach’s alpha of 0.76, revealing good internal consistency.

#### Kessler psychological distress scale (K10)

The K10 scale, designed by [29], consists of 10 items, each with a five-point Likert scale, describing different emotional states presented in section C. The scale can be used as a quick screening tool to determine the severity of discomfort. Each item’s response ranges from “all of the time” (score 4) to “none of the time” (score 0) with higher scores indicating more psychological distress. In the original Kessler Psychological Distress Scale (K10), the Cronbach’s alpha, a statistical measure used to assess the internal consistency reliability of the scale, was reported to be high at 0.93, indicating excellent reliability. With a Cronbach’s alpha of 0.80, the Psychological Distress scale demonstrates good internal consistency in the current research.

#### Research Self-efficacy scale (RSS/RSES)

It was designed by Büyüköztürk et al., 2011 [30] and consists of 30 items each with a five-point Likert scale designed to identify researchers’ self-efficacy in conducting their research in section D. RSS focuses on assessing one’s beliefs about his or her capacity to complete core tasks successfully in all analytical levels of research, from defining the issue to summarizing the results. Each item’s response ranges from “do not agree completely” (score 0) to “agree completely” (score 4), with higher scores indicating a higher perception of research self-efficacy. In the original Research Self-Efficacy (RSE) Scale by Büyüköztürk et al. (2011) demonstrated factor loadings for its items ranging from 0.48 to 0.66. The first factor of the scale had an eigenvalue of 5.50, which was more than four times greater than that of the second factor (1.2), accounting for 31% of the total variance. The scale also showed high reliability, with a Cronbach’s alpha of 0.87 and a Spearman-Brown split-half reliability coefficient of 0.85. In addition, current study, the Research Self-efficacy scale exhibits an even higher Cronbach’s alpha value of 0.87, indicating very good internal consistency.

### Data collection

Data collection ensued after the approval from the Ethical Research Committee was granted (Approval No: 73). Data was collected over five months from December 2022 to May 2023. The authors employed hybrid data collection techniques, using electronic online forms and paper-based surveys. Regarding the online method, the authors pursued research groups and academic forums to distribute an online form using Google Forms™ with the data collection tools. Next, the authors shared the link with the participants through their academic emails. A pilot study was carried out to examine the research transparency, clarity, and applicability of the study instruments. Those participants were not included in the final sample. The participants were provided with the study aim and objectives, and the consent form in the first two sections of the uploaded form. Appending the participants’ signatures reflects their voluntary engagement. Only one response per nursing scholar was allowed.

### Ethical concerns

The approval was granted from the Ethical Research Committee at the Faculty of Nursing, Damanhur University in El-Behera, Egypt (RES: 73). All volunteers gave their informed consent after receiving thorough information about the goals, dangers, and advantages of the study, which was included in the first page of the questionnaire. Anonymization and secure data storage procedures were used to scrupulously protect confidentiality and data privacy. Any conflicts of interest were declared, and the study was conducted by all applicable ethical standards and rules of conduct.

### Statistical analysis

The gathered data underwent organization, tabulation, and statistical analysis using SPSS software, version 25 (Statistical Package for the Social Sciences). A stepwise model-building approach was used to develop the multiple linear regression model predicting psychological distress. Spearman’s Correlation Coefficient was used to examine relationships between psychological distress and continuous variables. Only variables showing significant correlations (*p* ≤ 0.05) were considered for inclusion in the regression model. Independent samples t-tests and one-way ANOVA were used to compare psychological distress across categorical variables (e.g., gender, education level, experience in publishing, and type of publication). Variables demonstrating statistically significant differences (*p* ≤ 0.05) were included in the model. A hierarchical entry method was used for the multiple linear regression model, with variables entered in blocks based on theoretical relevance and statistical significance. Research Self-Efficacy and AI Concerns were entered first, followed by demographic factors (age, gender, years of experience), academic factors (education level, number of published articles, and type of publication), and experience in publishing as the final block.

## Results

### Study demographics and differences in study variables among academic staff

A total of 1,618 nursing scholars voluntarily participated in the study, with 1,494 valid responses, yielding a response rate of 92.34%. Table 1 presents the demographic characteristics of the participants. The sample consisted of 448 males (30%) and 1,046 females (70%), with a mean age of 39.17 years (SD = 6.79). Participants had an average of 11.96 years (SD = 5.79) of academic experience, and 87.9% had experience in publishing. The geographical distribution of participants was as follows: Delta and Lower Egypt: 30.9%, Central Region: 21.1%, Western and Eastern Regions: 30.7%, and Upper Egypt: 17.3%.

According to Table 1, there was a significant difference in psychological distress about gender, education, experience in publishing, and publication type. A significant difference in research self-efficacy regarding gender, education, experience in publishing, and publication type of participants. Concerns about AI varied significantly according to gender, education, experience in publishing, and publication type.

**Table 1:** Sample demographics and differences in study variables (*N = 1494)*

Characteristic	Category	No. (%)	Psychological distress		Research self-efficacy		Concerns toward AI	
			M (SD)	t/F (P)	M (SD)	t/F (P)	M (SD)	t/F (P)
Gender	Male	448 (30)	3.51 (0.48)	5.949 (< 0.001)	2.90 (0.53)	4.785 (< 0.001)	3.20 (0.46)	2.243 (0.036)
	Female	1046 (70)	3.34 (0.52)		3.05 (0.53)		3.25 (0.39)	
Study Discipline	Women’s and children’s health	522 (34.9)	3.40 (0.49)	0.321 (0.725)	3.99 (0.52)	0.553 (0.575)	3.22 (0.40)	0.669 (0.512)
	Medical, surgical& critical	757 (50.7)	3.38 (0.52)		3.01 (0.54)		3.24 (0.42)	
	Community& mental health	215 (14.4)	3.39 (0.55)		3.04 (0.55)		3.25 (0.42)	
Education	BSC	63 (4.2)	2.94 (0.49)	21.920 (< 0.001)	2.92 (0.67)	2.689 (0.045)	2.99 (0.22)	22.6 (< 0.001)
	Master	152(10.2)	3.54 (0.63)		3.0 (0.55)		3.20 (0.44)	
	PhD	535 (35.8)	3.42 (0.53)		3.05 (0.50)		3.33 (0.40)	
	Post Doc	744 (49.8)	3.38 (0.46)		2.98 (0.54)		3.19 (0.41)	
University Location	Center	315 (21.1)	3.37 (0.50)	0.532 (0.660)	3.06 (0.55)	2.170 (0.090)	3.25 (0.39)	1.539 (0.202)
	Delta	462 (30.9)	3.39 (0.53)		3.01 (0.54)		3.22 (0.41)	
	West and east	459 (30.7)	3.39 (0.52)		3.00 (0.55)		3.22 (0.42)	
	Upper Egypt	258 (17.3)	3.42 (0.50)		2.94 (0.46)		3.27 (0.41)	
Experience in publishing	No	181 (12.1)	3.19 (0.51)	5.599 (< 0.001)	2.87 (0.61)	3.546 (0.002)	2.94 (0.41)	10.142 (< 0.001)
	Yes	1313 (87.9)	3.42 (0.51)		3.02 (0.52)		3.27 (0.39)	
Publication Type	No	171 (11.4)	3.21 (0.49)	11.400 (< 0.001)	2.83 (0.59)	22.494 (< 0.001)	2.94 (0.42)	39.720 (< 0.001)
	National	479 (32.1)	3.38 (0.49)		3.16 (0.42)		3.32 (0.30)	
	International	79 (5.3)	3.57 (0.53)		2.96 (0.50)		3.17 (0.50)	
	Both	765 (51.2)	3.42 (0.52)		2.95 (0.57)		3.25 (0.43)	

According to Table 2, Psychological Distress is negatively correlated with Research Self-efficacy (*r* = -0.347**), suggesting that higher levels of Psychological Distress are associated with lower Research Self-efficacy. Psychological Distress shows weak positive correlations with Age (*r* = 0.106**) and Years of Experience (*r* = 0.138**), suggesting that as age and years of experience increase, so does psychological distress.

Research Self-efficacy exhibits weak negative correlations with Age (*r* = -0.074**) and Years of Experience (*r* = -0.111**), indicating that as individuals get older or gain more articles published, their Research Self-efficacy tends to decrease. On the other hand, Research Self-efficacy exhibits weak positive correlations with Years of Experience (*r* = 0.086**), indicating that as participants get more experience, their Research Self-efficacy tends to increase. Finally, concerns about AI have negligible correlations with the other variables.

### The interplay of research self-efficacy with concerns and psychological distress among academic staff

**Table 2:** Correlation between studied variables

Items		Mean (SD)	Research self-efficacy	Concerns about AI	Age	Years of experience	No published articles
Psychological Distress	r	3.39 (0.51)	− 0.347^**^	0.106^**^	0.138^**^	− 0.121^**^	0.113^**^
Research Self-efficacy	r	3 (0.54)	1	− 0.026	− 0.074^**^	0.086^**^	− 0.111^**^
Concerns about AI	r	3.23 (0.41)		1	0.005	0.001	0.061^*^
Age	r	39.17 (6.79)			1	0.204^**^	0.031
Years of Experience	r	11.96 (5.79)				1	0.154^**^
No Published Articles	r	8.59 (7.92)					1

R Pearson Correlation, **. Correlation is significant at the 0.01 level (2-tailed), *. Correlation is significant at the 0.05 level (2-tailed)

To identify variables that predict psychological distress, variables that showed a correlation with psychological distress were included in the regression model in Table 3. A multiple linear regression model was conducted to identify significant factors associated with psychological distress (Table 3). Research Self-Efficacy had the largest effect (B = -0.294, *p* < 0.001), indicating that higher self-efficacy was associated with lower psychological distress. Age and Years of Experience also had substantial impacts (B = 0.012, *p* < 0.001 and B = -0.016, *p* < 0.001, respectively), suggesting their meaningful roles in predicting psychological distress. The model explained approximately 21.4% of the variance in psychological distress (R² = 0.214) and was statistically significant (F = 56.885, *p* < 0.001).

In more detail, a one-unit increase in Research Self-Efficacy was associated with a decrease in Psychological Distress by 0.294 units, while a one-unit increase in Concerns about AI was linked to an increase of 0.080 units. A one-year increase in Age was associated with a 0.012-unit increase in Psychological Distress, whereas being male (as opposed to female) was associated with a decrease of 0.211 units. A one-year increase in Years of Experience corresponded to a 0.016-unit decrease in Psychological Distress. Type of Article Published and Level of Education were not significant factors associated with Psychological Distress.

### Predicting psychological distress among academic staff

**Table 3:** Results of multiple linear regression analysis predicting psychological distress of the academic staff (*N* = 1494)

Model	B	t	*P*	95% CI
(Constant)	3.791	25.377	0.000	3.498–4.048
Research Self-efficacy	− 0.294	-12.638	0.000	− 0.339 - − 0.248
Concerns about AI	0.080	2.643	0.008	0.021 − 0.139
Age	0.012	6.263	0.000	0.008 − 0.015
Gender	− 0.211	-7.735	0.000	− 0.265 - − 0.158
Years of Experience	− 0.016	-6.391	0.000	− 0.021 - − 0.011
Level of education	0.028	1.431	0.153	− 0.010 − 0.066
No Published Articles	0.005	2.492	0.013	0.001 − 0.008
Type of Article Published (National vs. International vs. Both)	− 0.026	-1.754	0.080	− 0.054 − 0.003
Experience in publishing	0.276	5.525	0.000	0.178 − 0.374

*R* = 0.463; R^2^ = 0.214; Adjusted R^2^ = 0.209; F = 56.885^***^;

B = Coefficients, SE = standard error, β = standardized regression coefficient, 95% CI = 95% confidence

interval, *P* < 0.001

## Discussion

Although the intrusion state of AI has fascinated and intrigued people, many scholars have some concerns and worries and go as far as to foretell that AI may be destroying human civilizations [31, 32]. Our findings suggested psychological distress is negatively correlated with researchers’ self-efficacy while positively correlated with concerns regarding AI. Moreover, a significant difference in all the studied variables (psychological distress, research self-efficacy & concerns about AI) regarding gender, education, and experience in publishing and publication type of participants.

### The interplay of research self-efficacy with concerns and psychological distress among academic staff

The current study showed that research self-efficacy negatively correlated with the scholars’ concerns related to AI. This aligns with the findings of Livinți et al. (2021), who reported that research self-efficacy is strongly associated with interest in research, research identity, career intentions, research productivity, attitudes toward research, and mentoring experiences [24]. Their results suggest that fostering a strong research culture, addressing research anxieties, and developing mentoring relationships can enhance research self-efficacy. Additionally, Tiyuri et al. (2018) found a direct and significant relationship between research self-efficacy and academic performance among postgraduate students, emphasizing the need for targeted educational interventions to strengthen quantitative and computer skills [25].

This finding may be attributable to the fact that respondents with high self-efficacy typically have a positive attitude regarding AI, value their sense of autonomy, feel confident in understanding AI implications, and have control over the research process. Prior research attested that self-efficacious individuals usually felt less fear of not succeeding in accomplishing a given task. In this context, they might perceive that the AI world has opened up new possibilities for engaging in AI-driven technology [32, 33]. However, those with lower -self-efficacy may feel less confident, leading to increased concerns and reliance on external sources [16, 34, 35].

The exposure to AI gadgets and build-up of experience in academic work would foster the scholars’ sense of self-efficacy regarding the usefulness of technology [36]. This explanation is confirmed by the findings of the present study, which displayed that self-efficacy significantly positively correlated with years of experience and the number of published articles. This outcome could stem from the expectations and familiarity with AI of nursing scholars who frequently have lifelong experiences with AI in the pursuit of academic work [37].

The current study found that psychological distress is negatively correlated with research self-efficacy, highlighting a novel insight into the relationship between mental well-being and research confidence. While prior research by Fitriawan et al. (2023) examined the association between self-efficacy and psychological distress in the context of online learning readiness [26], their findings emphasized the need for targeted interventions such as online learning training, technical assistance, and psychological support to enhance self-efficacy and mitigate distress. Additionally, Fürtjes et al. (2023) found that self-efficacy directly influences anxiety levels, whereas stress strongly impacts both depression and anxiety [27, 38]. Their study suggests that interventions for anxiety should focus on enhancing self-efficacy, while depression management should prioritize stress reduction. Our findings extend this body of research by demonstrating that psychological distress can also undermine research self-efficacy, underscoring the importance of psychological support mechanisms in academic and research settings to foster resilience and confidence in research pursuits [39–41].

### Study demographics and differences in study variables among academic staff

As a result of analyzing the variables of interest according to socio-demographic and academic characteristics, the nursing scholars showed a significant positive correlation between psychological distress and age. Undoubtedly, age can play a crucial role in shaping the researchers’ concerns and anxiety toward AI. Wang et al. (2023) suggested that younger academics are more likely to be exposed to AI and associated technologies due to their growing up in the digital realm [42, 43]. In this sense, they could be more accustomed to AI applications, and hence capable of critically analyzing the ethical implications of AI, addressing potential biases and privacy concerns. On the other hand, elderly researchers might be less familiar with AI gadgets and systems, which would lead to more worries or anxiety owing to unfamiliarity or perceived hazards with AI, which could lead to more worries or anxiety owing to unfamiliarity.

Regarding the years of academic experience, the present study revealed that there was a negative correlation between psychological distress and years of experience. These results could be explained by the fact that researchers’ concerns about AI can vary depending on their level of research experience [44, 45]. In this case, researchers with more years of academic experience may have seen how AI has developed and changed over time. They might also be more aware of the ethical implications, limitations, and historical context of AI, which might affect their worries and concerns. This is exemplified by infringing upon human rights, bias, transparency, accountability, privacy, and informed consent [46]. Such encountered challenges or ethical dilemmas lead to more caution on the part of experienced researchers. In addition, researchers with more experience in the publishing process may be concerned about the potential loss of human control in areas like manuscript selection, peer review, and editorial decision-making [47, 48]. Thus, it’s worth mentioning that while age and experience can provide insights into researchers’ concerns, they are not the sole determinants. Individual differences, personal beliefs, and exposure to diverse perspectives also shape one’s concerns about AI.

### Predicting psychological distress among researchers among academic staff

Our regression analysis revealed that higher self-efficacy was associated with lower psychological distress, reinforcing the protective role of self-efficacy in mental well-being. Additionally, age and years of experience had significant effects on psychological distress, suggesting that older individuals and those with more experience may develop greater resilience against psychological distress. These findings align with Hosseini et al. (2023), who found that higher age and lower education were linked to greater self-efficacy and improved coping styles [28]. Their study also highlighted that individuals with higher self-efficacy experienced better quality of life (QoL), while maladaptive coping strategies such as passive reacting and emotional expression were associated with increased psychological distress and reduced QoL. Kwak et al. (2022) avowed that self-efficacious researchers are more open to continuous learning and skill development [16], leveraging AI’s potential in their respective fields. They are more likely to engage in interdisciplinary research, fostering the integration of AI into diverse areas.

Additionally, our results are supported by Belloir et al. (2024), who demonstrated that general self-efficacy mediates the relationship between adverse childhood experiences and psychological distress, further underscoring the buffering role of self-efficacy against negative psychological outcomes [48]. Collectively, these findings emphasize the importance of fostering self-efficacy through targeted interventions, particularly among younger and less experienced individuals, to enhance coping mechanisms and mitigate psychological distress in research and professional settings [49].

In this regard, the responsible authorities and policymakers must create an inclusive environment for researchers to alleviate their stress, express concerns and foster collaborative problem-solving. Indeed, the AI adoption strategy would benefit the stakeholders by promoting information sharing, effort expectations and thereby enhancing the academicians’ overall mental well-being.

### Strengths& limitations

While the study employed a clustered sampling technique across 22 governmental nursing faculties in Egypt, ensuring representation from different geographical regions, the findings may still be context-specific and not fully generalizable to researchers from other academic disciplines. Future studies could explore perspectives on AI adoption in nursing education across diverse academic fields and cultural contexts to enhance generalizability. Longitudinal or experimental designs may provide deeper insights into the evolving perceptions and psychological impact of AI among nursing scholars. Additionally, this study is open to unobserved confounding, as not all information regarding AI use was collected. Factors such as individual experience with AI tools, institutional policies on AI integration, and prior exposure to AI-related training may have influenced participants’ perceptions but were not fully accounted for in the current analysis. Future research should consider these aspects to provide a more comprehensive understanding of AI-related concerns in nursing academia.

## Conclusion

This study underscores the critical role of research self-efficacy in mitigating psychological distress, highlighting its significance as a key protective factor. Additionally, age and years of experience, with older individuals experiencing slightly higher distress and greater experience, serve as a buffer. Gender differences were also observed, with male participants reporting lower distress levels. These insights contribute to a deeper understanding of the factors influencing psychological well-being in academic and research settings, guiding future strategies to foster resilience and mental well-being.

### Implications in nursing practice

In the era of AI, the implications of this study for nursing education are even more critical, as technological advancements continue to reshape research and clinical practice. The strong negative association between research self-efficacy and psychological distress highlights the need for nursing programs to integrate AI literacy, research mentorship, and adaptive learning strategies that build confidence in using AI-driven tools. Addressing concerns about AI through targeted education and training can help reduce anxiety and enhance students’ ability to engage with emerging technologies in research and practice.

Furthermore, stress management programs and psychological support systems should be embedded in nursing curricula to help students navigate the challenges of AI-driven healthcare environments. By fostering research self-efficacy and digital readiness, nursing education can better equip future nurses to leverage AI for evidence-based practice, innovation, and improved patient care while maintaining their psychological well-being.

Equally important, scholars are in a unique position to address ethical issues associated with the application and use of AI tools (such as drawing attention to the problematic aspects of using facial recognition software).

## Electronic supplementary material

Below is the link to the electronic supplementary material.

Supplementary Material 1

## Data Availability

Data are available on request from the corresponding author.
