# Supplementary material for: Unraveling research self-efficacy and concerns as factors associated with psychological distress among nursing scholars in the era of artificial intelligence: a multi-campus survey
Source: BMC Nurs. 2025 Jul 1;24:713. doi: 10.1186/s12912-025-03353-w (PMC12211384; doi:10.1186/s12912-025-03353-w)
Supplement: Supplementary file 1 — Supplementary Material 1 [file 12912_2025_3353_MOESM1_ESM.docx]

**Questionnaire for researcher**

**Dear participant**

**This questionnaire is designed to identify your concerns related to artificial intelligence. Rest assured that the data you provide will be treated with the utmost confidentiality and anonymity and will ONLY be used for research purposes. You also have the right to drop out of the study at any time without giving reasons. Thank you very much for your valuable participation.**

**Do you agree to participate in this study? Yes**  **No**

- Age : ……………….
- Sex : male female
- Number of working experience year : ……………………
- University affiliation: ………………………………….
- Level of education: Bachelor Master Doctoral
- Specialty: ………………………………………….
- Number of working experience year (example: 5) …………………. If less than one year write (1)
- Previously had experience in publishing: yes No
- No. of articles published (if none write: 0)
- Type of article publish was : None national international both
- Do you know about artificial intelligence tool that used in research? e.g. program sampling, in editing, reviewing...... Yes No If yes, enumerate one...........................

**Part 4 : psychometric tool designed to measure the concerns related to the use of artificial intelligence (AI) in research.**

| Items | All of the time  5 | Most of the time  4 | Some of the time  3 | A little of the time  2 | None of the time  1 |
| --- | --- | --- | --- | --- | --- |
| I have concerns related to the progress of artificial intelligence use in research. |  |  |  |  |  |
| I am concerned about keeping up with the advancements in artificial intelligence use in research. |  |  |  |  |  |
| The possibility of artificial intelligence use in research taking over tasks in my field makes me anxious. |  |  |  |  |  |
| I feel apprehensive about how artificial intelligence use in research may affect my decision-making abilities and professional judgment. |  |  |  |  |  |
| I am concerned that artificial intelligence use will devalue my expertise and knowledge in academic research. |  |  |  |  |  |
| I am not concerned about the dominance of artificial intelligence use in research. R |  |  |  |  |  |
| I am reassured of the helping role of artificial intelligence in research. R |  |  |  |  |  |
| I worry that artificial intelligence use in research will undermine human interaction. |  |  |  |  |  |
| The presence of artificial intelligence in academic research makes me concerned about job insecurity and increases stress and/or anxiety. |  |  |  |  |  |
| I feel anxious about the ethical implications of artificial intelligence use in research and its potential consequences for society. |  |  |  |  |  |
| The use of artificial intelligence in research makes me feel comfortable with my tasks. R |  |  |  |  |  |
| The use of artificial intelligence in research doesn’t bother me at all. R |  |  |  |  |  |
| I am anxious about the potential loss of job satisfaction and fulfillment due to advancements in artificial intelligence use in research. |  |  |  |  |  |
| I am concerned that artificial intelligence use in research will make as task oriented only. |  |  |  |  |  |
| The possibility of artificial intelligence replacing or outperforming me in my professional responsibilities in research makes me anxious. |  |  |  |  |  |
| I worry that artificial intelligence use in research will limit opportunities for professional growth and development in my field. |  |  |  |  |  |
| I feel apprehensive about the impact of artificial intelligence use in research on collaboration and teamwork within my work environment. |  |  |  |  |  |
| The integration of artificial intelligence in research makes me anxious about potential disruptions to my work-life balance. |  |  |  |  |  |
| I am worried that research roles will be developed using artificial intelligence in the future. |  |  |  |  |  |
| The use of artificial intelligence in research will help to optimize the significance of research. R |  |  |  |  |  |
